# Topical Ideal Sized Chitosan Dressing to Reduce Folliculitis After Microneedling, RF Microneedling, and Fractional CO_2_ Laser Resurfacing

**DOI:** 10.1111/jocd.70901

**Published:** 2026-06-04

**Authors:** Suyeon Lee, Soo Yeon Park, Wonseok Choi, Ji Yeoun Shin, Kyuho Yi

**Affiliations:** ^1^ Medical Research Inc. Seoul Korea; ^2^ Made‐Young Plastic Surgery Clinic Seoul Korea; ^3^ V Plastic Surgery Daegu Korea; ^4^ HEV Dermatology Clinic Seoul Korea; ^5^ You and I Clinic Seoul Korea

**Keywords:** acneiform eruption, chitosan, folliculitis, fractional CO_2_ laser, microneedling, RF microneedling, Sylfirm X

## Abstract

**Background and Objectives:**

Folliculitis, pustules, and acneiform eruptions are recognized early adverse events after microneedling, RF microneedling, and fractional laser resurfacing procedures that create microchannels or microthermal columns in the skin. Chitosan is a cationic biopolymer with film‐forming and antimicrobial properties and has been used as a wound dressing material.

**Study Design/Materials and Methods:**

We performed a single‐center retrospective cohort review of 324 consecutive patients undergoing microneedling (*n* = 140), RF microneedling (*n* = 121), or ablative fractional CO_2_ laser resurfacing (*n* = 63). Immediately after each procedure, 3 mL of a topical 2% chitosan formulation was applied as a postprocedure dressing. The primary outcome was clinician‐documented folliculitis/pustular eruption within 7 days following the procedure. Patient age and sex were not available in a complete analyzable format in the retrospective extract and therefore could not be summarized for the full cohort.

**Results:**

Two folliculitis/pustular events occurred among 324 patients (0.62%; 95% CI 0.17%–2.22%). After microneedling including injector, 1/140 patients (0.71%) developed a diffuse full‐face follicular papulopustular eruption. After RF microneedling, 1/121 patients (0.83%) developed a localized follicular eruption with only a few lesions. No folliculitis/pustules were recorded after ablative fractional CO_2_ laser resurfacing (0/63; 0%).

**Conclusions:**

In this retrospective cohort, standardized immediate postprocedure application of topical 2% chitosan was associated with a low observed frequency of folliculitis/pustular eruptions across microneedling, RF microneedling (Sylfirm X, Viol Medical, Korea), and fractional CO_2_ laser resurfacing (Beladona, Wontech, Korea), with variability observed between modalities. Controlled comparative studies with standardized demographic and device‐level data are needed to determine whether chitosan reduces risk relative to standard postprocedure dressings.

## 1 Introduction

Procedures that deliberately create microscopic injury patterns—mechanical microchannels (microneedling), thermomechanical coagulation columns (RF microneedling), or microscopic treatment zones with ablation and thermal coagulation (ablative fractional lasers)—are widely used to improve scars, texture, dyspigmentation, and photoaging. Although these modalities are generally safe, the early postprocedure period is characterized by transient barrier impairment and inflammation, which can precipitate follicular‐based pustular eruptions. In the literature, these events are variably recorded as “folliculitis,” “pustules,” “acne flare,” or “acneiform eruption,” and culture confirmation is uncommon, so the term often reflects a clinically observed syndrome rather than a microbiologically proven infection.

Fractional laser series and reviews identify acneiform eruptions as a relevant adverse event category. In a large retrospective analysis of 961 nonablative fractional laser treatments, acneiform eruptions were reported in 1.87% of treatments [1]. In a review of 373 fractionated carbon dioxide laser treatments, acneiform breakout occurred in 3.5% of treatments [2]. Broader reviews of fractional resurfacing complications and fractional CO_2_ (Beladona, Wontech, Korea) resurfacing likewise emphasize acneiform eruptions and folliculitis‐like reactions as clinically important early adverse events [3, 4]. Additional reviews of fractional resurfacing have also discussed expected adverse‐effect profiles and recovery patterns after ablative and nonablative platforms [5, 6].

Microneedling is often considered a low‐risk intervention; nevertheless, systematic and practical reviews note that pustules, acne flare, and other unexpected reactions can occur depending on patient susceptibility, treatment parameters, and postprocedure care [7, 8, 9]. For RF microneedling, temporary aggravation of acne vulgaris or folliculitis has been reported in clinical series, and more recent reviews confirm that post‐treatment inflammatory eruptions remain relevant to safety counseling [10, 11].

Because these pustular eruptions can negatively affect patient satisfaction, prolong downtime, and raise concerns about infection or scarring, prevention strategies are clinically valuable. Postprocedure topical care is a modifiable factor that can alter hydration, occlusion, and microbial load. Classic occlusive ointments support barrier recovery but may increase follicular occlusion in sebaceous or acne‐prone patients, potentially contributing to acneiform flares. Therefore, an ideal postprocedure dressing would support healing while minimizing follicular occlusion and microbial proliferation.

Chitosan is a deacetylated derivative of chitin and is widely discussed in wound care as a biocompatible, biodegradable polymer with film‐forming properties. Reviews describe intrinsic antimicrobial effects and wound‐supportive behavior, and chitosan‐based dressings have been investigated across multiple wound contexts [12, 13, 14, 15, 16]. Mechanistically, chitosan's cationic charge may disrupt microbial membranes and influence biofilm formation, with activity dependent on molecular weight, degree of deacetylation, and formulation [13, 14, 15, 16]. We hypothesized that immediate postprocedure application of topical 2% chitosan as a semipermeable dressing could be associated with a low frequency of folliculitis/pustular eruptions after microneedling, RF microneedling, and fractional CO_2_ laser resurfacing procedures. Here, we report a 324‐patient retrospective cohort treated with microneedling, RF microneedling, or ablative fractional CO_2_ laser resurfacing followed by standardized topical chitosan application.

## 2 Materials and Methods

### 2.1 Study Design and Population

This was a retrospective cohort study based on chart review in a single‐center clinical setting. Consecutive patients undergoing one of three procedures—microneedling, RF microneedling, or ablative fractional CO_2_ laser (Beladona, Wontech, Korea) resurfacing were included, provided that standardized topical 2% chitosan was applied immediately postprocedure.

Baseline demographic variables were requested during peer review; however, patient age and sex were not available in a complete analyzable format across the retrospective adverse‐event surveillance extract used for this study. Accordingly, these variables could not be summarized reliably for the entire cohort. This represents a significant limitation, as patient demographic characteristics may influence the risk of acneiform or follicular eruptions and could not be accounted for in this analysis.

### 2.2 Procedures and Grouping

Patients were treated with microneedling (*n* = 140), RF microneedling (*n* = 121), or ablative fractional CO_2_ laser resurfacing (*n* = 63). Procedural parameters (needle depth, RF energy settings, laser density/energy) were selected according to clinical indication and were not standardized for study purposes.

### 2.3 Device Platform Clarification

The analysis was performed at the level of treatment modality rather than individual device platform. Within the RF microneedling (Sylfirm X, Viol Medical, Korea) and ablative fractional CO_2_ subgroups, machine‐level information was not recorded in a sufficiently standardized manner in the retrospective chart extract; therefore, we could not confirm that the same platform was used for every participant within a subgroup, and device‐specific comparisons were not performed.

### 2.4 Postprocedure Dressing Protocol

Immediately after completion of the procedure and routine cleansing, 3 mL of a topical 2% chitosan formulation was applied to the treated region as a uniform thin layer. This application was performed once immediately postprocedure as part of the in‐clinic protocol. Postprocedure home‐care regimens were not standardized within the retrospective dataset and were not consistently documented, and therefore could not be analyzed in this study.

### 2.5 Outcome Definition

The primary outcome was clinician‐documented folliculitis/pustular eruption during the early postprocedure period, defined as within 7 days following the procedure. Because chart terminology varied, the outcome included events recorded as folliculitis, pustules, acneiform eruption, or acne flare, provided that follicular‐based papulopustular lesions were described on the treated area. Culture confirmation was not routinely performed and was not required for inclusion as an event.

### 2.6 Statistical Analysis

Observed frequencies were reported as counts and proportions for the overall cohort and by procedure subgroup. Wilson 95% confidence intervals (CIs) were calculated for descriptive context given low event rates.

## 3 Results

**FIGURE 1:**
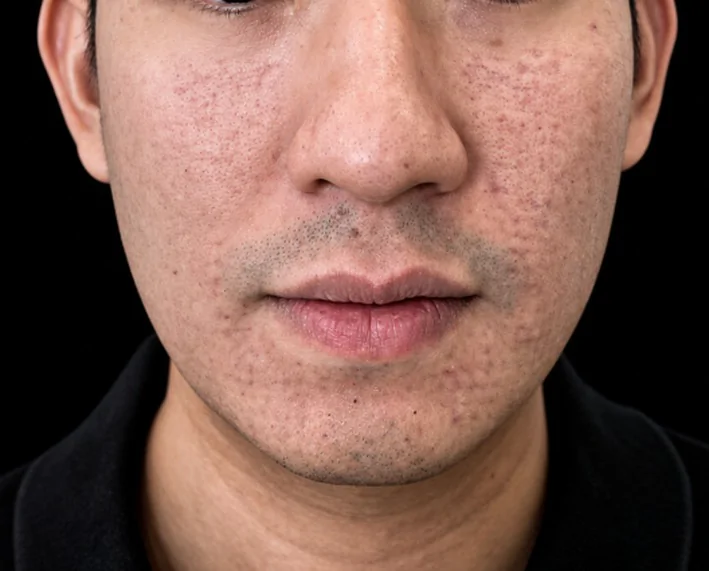
Injector gun (Rejumate, Pharmaresearch Inc., Korea) treatment with topical 2% chitosan postprocedure dressing on right side and no postprocedure on the left side. Eight hours after the right side had lesser downtime without diffuse pustular eruption.

A total of 324 patients were included: microneedling (*n* = 140), RF microneedling (*n* = 121, (Sylfirm X, Viol Medical, Korea)), and ablative fractional CO_2_ laser resurfacing (*n* = 63). Table 1 summarizes procedure distribution and observed folliculitis/pustule events across the complete cohort. Two early postprocedure folliculitis/pustular events were recorded, corresponding to an overall observed frequency of 0.62% (2/324; 95% CI 0.17%–2.22%). Representative clinical outcomes following microneedling and RF microneedling are shown in Figure 1.

**TABLE 1:** Procedure distribution and observed folliculitis/pustule events (complete cohort dataset).

Procedure modality	Patients (*n*)	Folliculitis/pustule events (*n*)	Observed event rate (%)
Microneedling	140	1	0.71
RF microneedling	121	1	0.83
Ablative fractional laser	63	0	0.00
Total	324	2	0.62

Age and sex were not available in a complete analyzable format across the retrospective extract and therefore are not tabulated for the full cohort.

In the microneedling group, 1/140 patients (0.71%; 95% CI 0.13%–3.93%) developed a diffuse full‐face follicular papulopustular eruption. The chart described a monomorphic, diffuse facial eruption rather than a few isolated lesions, making this the more extensive of the two recorded events. Standardized information on baseline acne history, sebaceous activity, and sex was not available for this case.

In the RF microneedling group, 1/121 patients (0.83%; 95% CI 0.15%–4.53%) developed a localized follicular eruption characterized by only a few lesions in the treated area. No folliculitis/pustules were documented in the ablative fractional CO_2_ laser group (0/63; 0%; 95% CI 0%–5.75%). Event‐level descriptors are summarized in Table 2.

**TABLE 2:** Event‐level dataset (all recorded folliculitis/pustule events).

Event ID	Modality	Extent described clinically	*n*
1	Microneedling	Diffuse full‐face follicular papulopustular eruption	1
2	RF microneedling (Sylfirm X, Viol Medical, Korea)	Localized folliculitis (“few lesions”)	1

No microbiologic confirmation was routinely obtained, and no standardized baseline acne/seborrhea phenotype data were available for subgroup analysis in the two event cases.

## 4 Discussion

Follicular papulopustular eruptions remain an important early adverse‐event category after procedures that transiently disrupt the epidermal barrier. Published fractional laser series and reviews consistently describe acneiform eruption or folliculitis‐like reactions among recognized postprocedure complications [1, 2, 3, 4, 5, 6]. Microneedling is widely regarded as safe, but systematic and practical reviews emphasize that pustules, acne flare, and other inflammatory reactions can still occur under certain clinical conditions [7, 8, 9]. RF microneedling literature similarly supports counseling patients about transient inflammatory eruptions, including acne aggravation or folliculitis‐like reactions [10, 11].

The clinically relevant observation in our cohort is the difference between modalities rather than a single pooled estimate. One patient in the microneedling subgroup developed a diffuse full‐face eruption, whereas the patient in the RF microneedling subgroup developed only a few localized lesions. This pattern suggests that postprocedure follicular reactions may differ in extent even under the same dressing protocol. Host factors such as acne susceptibility, sebaceous activity, or prior folliculitis may have contributed, but those variables were not documented in a standardized manner and therefore cannot be analyzed here. Representative postprocedure recovery patterns are illustrated in Figures 1.

The biological rationale for chitosan as a postprocedure dressing is supported by wound‐care literature. Chitosan is described as a film‐forming biomaterial that can act as a semipermeable dressing while also exhibiting antimicrobial and wound‐supportive behavior [12, 13, 14, 15, 16]. Its cationic character may interact with negatively charged microbial cell membranes, inhibit microbial growth, and influence biofilm formation [13, 14, 15, 16]. In skin that has undergone microneedling, RF microneedling (Sylfirm X, Viol Medical, Korea), or fractional laser resurfacing, these properties provide a plausible biological rationale for compatibility with a low frequency of clinically observed follicular pustular events.

A second hypothesis relates to occlusion. Traditional petrolatum‐based or heavier occlusive regimens can be effective for barrier support, but in acne‐prone patients they may also increase follicular occlusion. A thin film‐forming chitosan dressing may provide barrier assistance with a different occlusive profile than thick ointments. The present study cannot test that mechanism directly, but it offers a clinically relevant rationale for future comparator trials.

Several limitations prevent causal inference. The retrospective design and absence of a contemporaneous control group do not allow determination of whether chitosan reduced risk relative to standard postprocedure care. Outcome capture depended on chart documentation and may underestimate mild eruptions managed outside the clinic. The event definition grouped clinically similar entities without routine microbiologic confirmation. Importantly, demographic variables such as age and sex were not available in a complete analyzable format, precluding assessment of known risk modifiers for acneiform eruptions and limiting interpretation of the findings. Procedural parameters were individualized, and the analysis was performed by treatment modality rather than by device model because machine‐level data were not standardized. Finally, additional patient‐level risk factors such as baseline acne history, sebaceous activity, or prior folliculitis were not available for analysis.

Within these constraints, the findings provide preliminary clinical support that immediate topical 2% chitosan after microneedling, RF microneedling, and fractional CO_2_ laser resurfacing is feasible and associated with a low recorded frequency of folliculitis/pustular events in routine practice. The appropriate next step is a prospective controlled study comparing topical chitosan with common postprocedure regimens, using standardized follow‐up, explicit eruption grading, device‐level reporting, and stratification by acne risk profile.

## 5 Conclusions

In a retrospective cohort of 324 patients undergoing microneedling, RF microneedling, or ablative fractional CO_2_ laser resurfacing, immediate application of 3 mL topical 2% chitosan was associated with a low observed frequency of folliculitis/pustular eruptions. These findings are descriptive and hypothesis‐generating and support feasibility; however, controlled prospective studies are required to determine whether topical chitosan reduces folliculitis risk compared with standard postprocedure dressings.

## Author Contributions

Conceptualization: Kyuho Yi. Methodology: Wonseok Choi, Ji Yeoun Shin. Investigation: Wonseok Choi, Ji Yeoun Shin. Data curation: Suyeon Lee, Soo Yeon Park. Visualization: Suyeon Lee, Soo Yeon Park. Writing – original draft: Wonseok Choi, Ji Yeoun Shin, Kyuho Yi. Writing – review and editing: Wonseok Choi, Ji Yeoun Shin, Kyuho Yi. Supervision: Kyuho Yi.

## Funding

The authors have nothing to report.

## Consent

Written informed consent was obtained from all participants prior to the procedure. Patients provided written consent for the use of their clinical information and photographs for research and publication purposes.

## Conflicts of Interest

The authors declare no conflicts of interest.

## Data Availability

The data that support the findings of this study are available from the corresponding author upon reasonable request.
