# Correction to “Topical Ideal Sized Chitosan Dressing to Reduce Folliculitis After Microneedling, RF Microneedling, and Fractional CO_2_ Laser Resurfacing”

**DOI:** 10.1111/jocd.71143

**Published:** 2026-08-19

S. Lee , S. Y. Park , W. Choi , J. Y. Shin , K. Yi , “Topical Ideal Sized Chitosan Dressing to Reduce Folliculitis After Microneedling, RF Microneedling, and Fractional CO_2_ Laser Resurfacing,” Journal of Cosmetic Dermatology (2026), 10.1111/jocd.70901.

The name of the fourth author was incorrect and has been updated from Jiyeon Shin to Ji Yeoun Shin.

The online version of this article has been corrected accordingly.

We apologize for this error.

